# Impact of a balloon-in-basket pulsed field ablation catheter on oesophageal temperature changes during pulmonary vein isolation

**DOI:** 10.1093/europace/euag115

**Published:** 2026-06-30

**Authors:** Julia Vogler, Roman Mamaev, Jan-Per Wenzel, Charlotte Eitel, Sorin Stefan Popescu, Zeynap Gizem Demirtakan, Tugba Aktemur Özalp, Julius Nikorowitsch, Sascha Hatahet, Behnam Subin, Samuel Reincke, Anna Traub, Engin Yaman, Mirco Küchler, Karl-Heinz Kuck, Roland Richard Tilz

**Affiliations:** University Heart Centre Lübeck, Department of Rhythmology, University Hospital Schleswig-Holstein, Ratzeburger Allee 160, Campus Lübeck 23538, Germany; Department of Cardiology and Internal Intensive Care Medicine, Asklepios Hospital St.Georg, Lohmühlenstr. 5, Hamburg 20099, Germany; Die Sektion Medizin, Universität zu Lübeck, Lübeck 23538, Germany; University Heart Centre Lübeck, Department of Rhythmology, University Hospital Schleswig-Holstein, Ratzeburger Allee 160, Campus Lübeck 23538, Germany; University Heart Centre Lübeck, Department of Rhythmology, University Hospital Schleswig-Holstein, Ratzeburger Allee 160, Campus Lübeck 23538, Germany; German Centre for Cardiovascular Research (DZHK), Partner Site Hamburg/Kiel/Lübeck, Lübeck, Germany; University Heart Centre Lübeck, Department of Rhythmology, University Hospital Schleswig-Holstein, Ratzeburger Allee 160, Campus Lübeck 23538, Germany; University Heart Centre Lübeck, Department of Rhythmology, University Hospital Schleswig-Holstein, Ratzeburger Allee 160, Campus Lübeck 23538, Germany; University Heart Centre Lübeck, Department of Rhythmology, University Hospital Schleswig-Holstein, Ratzeburger Allee 160, Campus Lübeck 23538, Germany; University Heart Centre Lübeck, Department of Rhythmology, University Hospital Schleswig-Holstein, Ratzeburger Allee 160, Campus Lübeck 23538, Germany; University Heart Centre Lübeck, Department of Rhythmology, University Hospital Schleswig-Holstein, Ratzeburger Allee 160, Campus Lübeck 23538, Germany; German Centre for Cardiovascular Research (DZHK), Partner Site Hamburg/Kiel/Lübeck, Lübeck, Germany; University Heart Centre Lübeck, Department of Rhythmology, University Hospital Schleswig-Holstein, Ratzeburger Allee 160, Campus Lübeck 23538, Germany; University Heart Centre Lübeck, Department of Rhythmology, University Hospital Schleswig-Holstein, Ratzeburger Allee 160, Campus Lübeck 23538, Germany; University Heart Centre Lübeck, Department of Rhythmology, University Hospital Schleswig-Holstein, Ratzeburger Allee 160, Campus Lübeck 23538, Germany; University Heart Centre Lübeck, Department of Rhythmology, University Hospital Schleswig-Holstein, Ratzeburger Allee 160, Campus Lübeck 23538, Germany; University Heart Centre Lübeck, Department of Rhythmology, University Hospital Schleswig-Holstein, Ratzeburger Allee 160, Campus Lübeck 23538, Germany; University Heart Centre Lübeck, Department of Rhythmology, University Hospital Schleswig-Holstein, Ratzeburger Allee 160, Campus Lübeck 23538, Germany; University Heart Centre Lübeck, Department of Rhythmology, University Hospital Schleswig-Holstein, Ratzeburger Allee 160, Campus Lübeck 23538, Germany; University Heart Centre Lübeck, Department of Rhythmology, University Hospital Schleswig-Holstein, Ratzeburger Allee 160, Campus Lübeck 23538, Germany; German Centre for Cardiovascular Research (DZHK), Partner Site Hamburg/Kiel/Lübeck, Lübeck, Germany

**Keywords:** Atrial fibrillation, Oesophageal temperature, Oesophageal thermal injury, Pulsed field ablation

## Introduction

Pulsed field ablation (PFA) has emerged as a novel energy modality for catheter ablation of atrial fibrillation (AF). Unlike thermal ablation techniques, PFA offers tissue specificity, potentially reducing fatal complications such as atrio-oesophageal fistulas. However, data on PFA's effect on the oesophagus remain limited. Preclinical studies demonstrate that supratherapeutic PFA induces acute oesophageal lesions that resolve over time, while clinical studies report no oesophageal adverse events with current PFA catheters, although systematic assessment is lacking.^1^ Given that PFA lesions depend on electrical field strength, waveform, catheter design, and tissue proximity, safety data from one catheter may not apply to other designs. This subanalysis of the VOLT CE Mark Study aims to evaluate oesophageal temperature changes during pulmonary vein isolation (PVI) with a balloon-in-basket PFA catheter.

## Methods

Consecutive patients presenting for *de novo* PVI who were enrolled in the VOLT CE Mark Study at the University Heart Centre, Lübeck, were included in this analysis. The VOLT CE Mark Study and its procedural protocol have been described in detail.^2,3^ The study was approved by the local ethics committee. Baseline PFA therapy consisted of up to eight nominal applications per pulmonary vein (PV). Low-voltage therapy was mandated for the right PVs, if phrenic nerve capture could not be avoided by repositioning. No additional ablation beyond PVI was allowed.

Intraluminal oesophageal temperature (TESO) was continuously monitored with a 12-pole temperature probe (CIRCA S-CATH™, CIRCA Scientific, Inc.) before and after each PFA train. Post-ablation care was conducted according to the study protocol. Proton-pump inhibitors were administered twice daily until discharge and once daily for 6 weeks. No routine esophagoscopy was planned.

Data analysis was performed using IBM SPSS Statistics (Version 29.0.2.0). In case of normal distribution, data are presented as mean ± standard deviation, otherwise as median and interquartile ranges. Comparison of continuous data was performed using the student's t-test if normally distributed, otherwise with the Wilcoxon signed-rank test.

## Results

A total of 30 patients (median age 65 [60–71] years, 57% male, 50% PAF, LV function 55 ± 8%) underwent PVI with the balloon-in-basket catheter. Procedure, fluoroscopy, and LA dwell times were 78.2 ± 10.6 min, 10.0 ± 4.5 min, and 27.1 ± 8.5 min. A mean of 16.5 ± 2.0 PFA applications per subject (4.2 ± 1.1 per PV) was delivered.

**Figure 1:**
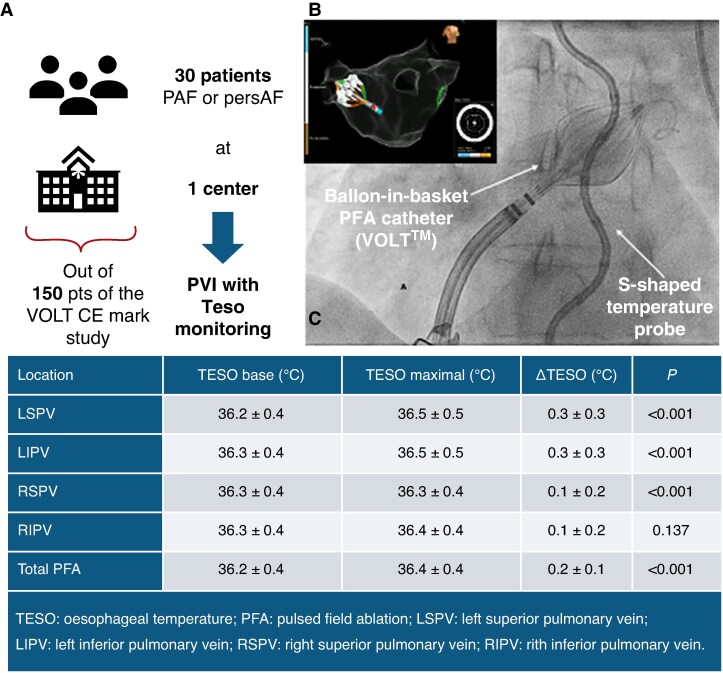
A: Study design. B: 3D mapping (Abbott EnSite™ X) with the VOLT™ PFA catheter in the right inferior pulmonary vein. C: Fluoroscopy of the left superior pulmonary vein in left anterior oblique (LAO) projection during PFA-PVI using the VOLT™ PFA catheter. Table at the bottom: Mean intraluminal oesophageal temperature changes during pulmonary vein isolation using a new balloon-in-basket PFA system (*n* = 29).

Positioning of an oesophageal temperature probe was feasible in 29/30 patients. The oesophagus passed near the left PVs in 20/29 (69.0%), near the right PVs in 2/29 (6.9%), and directly behind the posterior wall in 7/29 (23.3%) patients. Mean TESO increased by 0.2 ± 0.1°C (*P* < 0.001), with no change >1.0°C and a maximum temperature of 37.8°C (*Figure 1)*. Temperature changes were more pronounced during ablation of the left PVs compared to ablation of the right PVs (0.3 ± 0.3 vs. 0.1 ± 0.2). When comparing a left-sided, a right-sided, and a posterior course of the oesophagus (in relation to the PVs), no temperature changes were observed for a posterior course of the oesophagus with ablation remote from the probe, while an increase of 0.28°C was detected when ablating the left PVs (on-the-probe) in a left-sided course compared to 0.05°C during ablation of the right PVs. A Δ TESO of 0.38°C (right PVs) and 0.27°C was observed in the two patients with a right-sided oesophagus course.

No patient reported symptoms of oesophageal injury during a follow-up of 6 months, precluding the need for esophagoscopy. No major complications occurred.

## Discussion

This is the first study to evaluate oesophageal temperature changes during PVI with a balloon-in-basket PFA catheter. The main findings can be summarized as follows: (i) A balloon-in-basket PFA PVI resulted in minimal TESO changes, indicating that PFA is not a strictly non-thermal energy. (ii) Ablation in close proximity to the oesophagus resulted in detectable intraluminal TESO changes. (iii) No symptoms suggestive of oesophageal injury were observed.

While PFA is regarded as nonthermal energy with a superior safety profile compared to thermal ablation, objective measurements and mathematical models indicate significant temperature increases can occur at the catheter tip during ablation.^4^ Our findings support the presence of thermal effects with PFA. While we observed no TESO changes during ablation remote from the oesophagus, a median TESO change of 0.3°C was recorded during ablation of PVs adjacent to the oesophagus. From a clinical perspective, these TESO changes were mild (<1.0°C) and probably clinically irrelevant. TESO changes with the balloon-in-basket PFA catheter were less pronounced than those previously reported with a pentaspline catheter.^5^ This difference may be attributed to variations in catheter design and ablation strategy, as our pentaspline approach included wide antral PVI extending to the posterior wall, whereas the present study performed PVI alone. Thus, the risk of severe oesophageal complications appears minimal, consistent with other PFA catheters. However, whether this also applies for persistent AF ablation, when multiple PFA applications are delivered, remains uncertain.

Clinical data show no oesophageal injuries among >2000 treated patients, although systematic oesophageal assessment remains limited.^5–10^ Most studies used endoscopy, which is insensitive for PFA-induced adventitial lesions, while limited cardiac MRI studies identified no post-PFA oesophageal lesions.^1,6,10^ Only four trials employed oesophageal temperature monitoring, with one reporting low-level heating (<39°C) during posterior wall ablation, consistent with our findings.^5,8,9^

While our data align with clinical evidence showing negligible oesophageal impact compared to thermal ablation at the same level of efficacy, they challenge the assumption that PFA is entirely non-thermal. Given that each PFA system employs proprietary waveforms and protocols and that limited thermal effects do occur, the safety profile of one system cannot be extrapolated to other platforms.

## Limitations

Our analysis is limited by a small sample size and operator-dependent PFA application numbers, which may affect the risk of local heating. Without routine esophagoscopy or cardiac imaging, mild asymptomatic oesophageal injuries may have been undiagnosed. Additionally, current temperature probes may inadequately capture PFA's rapid temperature changes.

## Conclusion

PVI delivered via a balloon-in-basket PFA catheter results in minor oesophageal temperature increases of questionable clinical relevance. Continued vigilance and systematic safety assessment remain essential as new PFA technologies emerge.

## Ethics approval

The present study was approved by the local ethics committee (file number: 2022–598) and was performed in accordance with the ethical standards laid down in the 1964 Declaration of Helsinki and its later amendments. The VOLT CE Mark study was sponsored by Abbott.

## Consent for publication

The manuscript “*Impact of a new balloon-in-basket pulsed field ablation system on esophageal temperature changes during pulmonary vein isolation*” is not under consideration for publication elsewhere. None of the paper’s contents has been previously published. All authors have read and approved the manuscript.

## Data Availability

Data are available upon reasonable request at the corresponding author.

## References

[1] Nies M, Watanabe K, Kawamura I, Koruth JS. Endocardial pulsed field ablation and the oesophagus: are atrio-oesophageal fistulas now history? Arrhythm Electrophysiol Rev 2024;13:e02. 38544810 10.15420/aer.2023.16 PMC10964285

[2] Sanders P, Healy S, Emami M, Kotschet E, Miller A, Kalman JM. Initial clinical experience with the balloon-in-basket pulsed field ablation system: acute results of the VOLT CE mark feasibility study. Europace 2024;26:euae118. 38701222 10.1093/europace/euae118 PMC11098042

[3] Tilz RR, Chierchia GB, Gunawardene M, Sanders P, Haqqani H, Kalman J et al Safety and effectiveness of the first balloon-in-basket pulsed field ablation system for the treatment of atrial fibrillation: VOLT CE mark study 6-month results. Europace 2025;27:euaf072. 40163671 10.1093/europace/euaf072 PMC12036658

[4] van Veldhuisen E, Vogel JA, Klaessens JH, Verdaasdonk RM. Thermal effects of irreversible electroporation. In: Irreversible Electroporation in Clinical Practice. Cham: Springer; 2018.

[5] Kirstein B, Heeger CH, Vogler J, Eitel C, Feher M, Phan HL et al Impact of pulsed field ablation on intraluminal esophageal temperature. J Cardiovasc Electrophysiol 2024;35:78–85. 37942843 10.1111/jce.16096

[6] Cochet H, Nakatani Y, Sridi-Cheniti S, Cheniti G, Ramirez FD, Nakashima T et al Pulsed field ablation selectively spares the oesophagus during pulmonary vein isolation for atrial fibrillation. Europace 2021;23:1391–9. 33961027 10.1093/europace/euab090 PMC8427383

[7] Duytschaever M, De Potter T, Grimaldi M, Anic A, Vijgen J, Neuzil P et al Paroxysmal atrial fibrillation ablation using a novel Variable-loop biphasic pulsed field ablation catheter integrated with a 3-dimensional mapping system: 1-year outcomes of the multicenter inspIRE study. Circ Arrhythm Electrophysiol 2023;16:e011780. 36735937 10.1161/CIRCEP.122.011780 PMC10026968

[8] Reddy VY, Anter E, Rackauskas G, Peichl P, Koruth JS, Petru J et al Lattice-tip focal ablation catheter that toggles between radiofrequency and pulsed field energy to treat atrial fibrillation: a first-in-human trial. Circ Arrhythm Electrophysiol 2020;13:e008718. 32383391 10.1161/CIRCEP.120.008718

[9] Schmidt B, Bordignon S, Tohoku S, Chen S, Bologna F, Urbanek L et al 5S study: safe and simple single shot pulmonary vein isolation with pulsed field ablation using sedation. Circ Arrhythm Electrophysiol 2022;15:e010817. 35617232 10.1161/CIRCEP.121.010817

[10] Reddy VY, Dukkipati SR, Neuzil P, Anic A, Petru J, Funasako M et al Pulsed field ablation of paroxysmal atrial fibrillation: 1-year outcomes of IMPULSE, PEFCAT, and PEFCAT II. JACC Clin Electrophysiol 2021;7:614–27. 33933412 10.1016/j.jacep.2021.02.014

